# Association between musculoskeletal pain and exposures to awkward postures during work: a compositional analysis approach

**DOI:** 10.1093/annweh/wxae027

**Published:** 2024-04-11

**Authors:** Fredrik Klæboe Lohne, Kailiang Xu, Marius Steiro Fimland, Javier Palarea-Albaladejo, Skender Redzovic

**Affiliations:** Department of Neuromedicine and Movement Science, Faculty of Medicine and Health Sciences, Norwegian University of Science and Technology, Edvard Griegs gate 8, 7030, Trondheim, Norway; Department of Neuromedicine and Movement Science, Faculty of Medicine and Health Sciences, Norwegian University of Science and Technology, Edvard Griegs gate 8, 7030, Trondheim, Norway; Department of Orthopaedics, Beijing Miyun District Hospital, Yanguang Street, 383, 101500, Beijing, China; Department of Neuromedicine and Movement Science, Faculty of Medicine and Health Sciences, Norwegian University of Science and Technology, Edvard Griegs gate 8, 7030, Trondheim, Norway; Unicare Helsefort Rehabilitation Centre, Hysnesveien 11, 7112, Rissa, Norway; Department of Computer Sciences, Applied Mathematics and Statistics, University of Girona, Carrer Universitat de Girona 6, 17003, Girona, Spain; Department of Neuromedicine and Movement Science, Faculty of Medicine and Health Sciences, Norwegian University of Science and Technology, Edvard Griegs gate 8, 7030, Trondheim, Norway

**Keywords:** accelerometer, arm elevation, compositional data, low back pain, neck/shoulder pain, occupational health, trunk forward bending, upright postures

## Abstract

**Objectives:**

This study aimed to explore the association between arm elevation and neck/shoulder pain, and trunk forwarding bending and low back pain among home care workers.

**Methods:**

Home care workers (*N* = 116) from 11 home care units in Trondheim, Norway, filled in pain assessment and working hours questionnaire, and wore 3 accelerometers for up to 7 consecutive days. Work time was partitioned into upright awkward posture, nonawkward posture, and nonupright time, i.e. sitting. Within a compositional approach framework, posture time compositions were expressed in terms of log-ratio coordinates for statistical analysis and modeling. Poisson generalized linear mixed models were used to analyze the relationship between arm elevation in upright postures and neck/shoulder pain, and between trunk forward bending in upright postures and low back pain, respectively. Isotemporal substitution analysis was used to investigate the association of pain assessment with the reallocation of time spent in the different postures.

**Results:**

Time spent in awkward postures was modest, especially for the more extreme angles (60° and 90°). Adjusting for age, gender, and body mass index, our study suggested that the compositions of time spent by home care workers in awkward postures were significantly associated with pain assessment (*P* < 0.01). Isotemporal substitution analysis showed that reallocating 5 min from upright posture with arms elevated below to above 60° and 90° was associated with a 6.8% and 19.9% increase in the neck/shoulder pain score, respectively. Reallocating 5 min from a forward bending posture while upright below to above 30°, 60°, and 90° was associated with 1.8%, 3.5%, and 4.0% increase in low back pain, respectively.

**Conclusions:**

Although the exposure to awkward postures was modest, our results showed an association between increased time spent in awkward postures and an increase in neck/shoulder pain and low back pain in home care workers. As musculoskeletal pain is the leading cause of sickness absence, these findings suggest that home care units could benefit from re-organizing work to avoid excessive arm elevation and trunk forward bending in workers.

**What’s Important About This Paper?**

This study is the first to investigate the association between technically measured awkward postures at work and musculoskeletal pain at the end of the working day in home care workers. These results show a positive association between the time spent in forward leaning and arm elevation at work, and lower back and neck/shoulder pain, respectively. These findings can guide interventions and health management efforts in the home care sector.

## Background

In Norway, the current population of individuals aged 80 is projected to double by 2040 (Statistics Norway 2023a, 2023b). This elderly demographic often has limited mental and physical functioning, necessitating care from others. Consequently, home care workers, comprising nurses, occupational therapists, health assistants, physiotherapists, and social workers, play an indispensable role in providing care for the elderly and disabled population, now and in the future. Home care workers in Norway face a very high sick leave rate (11%), which is nearly double the national average (Norwegian Directorate of Health 2019; Statistics Norway 2022). Musculoskeletal pain in the neck/shoulder area and lower back is highly prevalent in the working population (Skovlund et al. 2020; Tjøsvoll et al. 2022) serving as the primary cause of sick leave (Andersen et al. 2016; Hallman et al. 2019). A recent study revealed that 36% of home care workers in Norway reported long-term neck/shoulder pain (NSP) and 34% reported long-term low back pain (LBP) in the past year (Tjøsvoll et al. 2022). Musculoskeletal pain reduces work ability (Hallman et al. 2019; Skovlund et al. 2020; Andersen et al. 2021) and constitutes a severe public health burden (Jin et al. 2020; Wærsted et al. 2020; Andersen et al. 2021).

Research conducted by Hittle et al. (2016) identified that moving clients in and out of chairs, toilets, and beds, along with repositioning, are the most frequent physical tasks encountered in patient care. These activities typically necessitate forward bending or arm elevation. Several studies have indicated that awkward postures at work, e.g. arm elevation and trunk forward bending, are risk factors for NSP and LBP, respectively (Svendsen et al. 2004; Van der Molen et al. 2017; Lunde et al. 2019; Wærsted et al. 2020). However, most studies have used self-reporting methods for assessing the exposure to awkward postures which suffer from subjectiveness and recall bias (Koch et al. 2016; Wærsted et al. 2020; Andersen et al. 2021). Studies using technical measurements have found conflicting results on the association between arm elevation and NSP. Some studies reported a positive relationship between arm elevation at work and NSP (Svendsen et al. 2004; Van der Molen et al. 2017; Wærsted et al. 2020), while others reported a negative association or no association (Luime et al. 2004; Koch et al. 2017; Merkus et al. 2021). To date, no studies have explored the association between technically measured arm elevation in upright postures at work and NSP in home care workers.

Studies also show conflicting results regarding the relationship between trunk forward bending and LBP (Swain et al. 2020), ranging from positive association (Tubach et al. 2002; Jansen et al. 2004) and no association (Lagersted-Olsen et al. 2016) to negative association (Villumsen et al. 2015). Recently, some studies have suggested that trunk forward bending in upright postures may be a risk factor for LBP (Palm et al. 2018; Andersen et al. 2021). However, a longitudinal study using accelerometers to measure postures found a positive association between LBP intensity and trunk forward bending >30° in upright postures, but not for >60°, in healthcare workers (Lunde et al. 2019). Hence, to the best of our knowledge, the relationship between trunk forward bending in upright postures at work and LBP intensity is still unclear.

Most studies assessing the association between awkward postures and NSP and LBP not only suffer from recall bias as a consequence of exposure measurements but also outcome. Pain is often assessed by asking participants to recall events of pain the previous month or year (Swain et al. 2020; Wærsted et al. 2020). Having participants assess the current pain, directly following the working day, reduces recall bias and offers the possibility of analyzing the daily exposure and corresponding pain.

Therefore, given the increasing number of the aged population, the health and workability of home care workers, and the high socioeconomic burden, it is of paramount importance to understand and promote suitable working conditions and reduce the occurrence and intensity of musculoskeletal pain in home care workers. This study aims to investigate whether there is an association between awkward postures during the entire workday and NSP and LBP, assessed at the end of the workday, in home care workers. The study seeks to test the following hypotheses: (i) there is a positive association between arm elevation in upright postures during the workday and NSP, and (ii) there is a positive association between trunk forward bending in upright postures during the workday and LBP.

## Methods

### Study population

Home care workers were recruited from 11 home care service units in Trondheim. All participants had paid employment and held a minimum of 50% full-time equivalent position in home care. To capture a normal home care workday, we excluded workers with physical disabilities or pregnancy. Furthermore, participants with a fever on the day of enrollment or skin allergy to plastic tapes were excluded.

All participants received written information about the research and provided informed written consent before the start of this study. Each participant was given a subject ID to protect their identity. The study was approved by the Regional Committees for Medical Research Ethics—Central Norway (No. 315556) and conducted in line with the Helsinki Declaration.

### Data collection

The data were collected by questionnaires, anthropometric measurements, and accelerometer measurements from August to November 2022. The data collection also served as the baseline data for a cluster randomized controlled trial—aiming at improving workers’ health by reorganizing the distribution of work tasks (Lohne et al. 2022).

Before beginning the accelerometer measurements and pain assessments, participants completed a questionnaire. They then wore accelerometers for up to 7 d. Concurrently, they filled out a daily pain questionnaire after each workday, regarding NSP and LBP. This approach resulted in data on exposure to awkward postures per day, with the corresponding daily pain score.

### Basic information and anthropometrics

Information regarding age, gender, occupation, and health status was collected by questionnaire. Participants’ weight was measured by a digital body weight scale and height by a wall-mounted SECA 206 measuring tape. Body mass index (BMI) was calculated based on the formula (BMI = weight/height^2^ (kg/m^2^)). Participants’ sick leave history was assessed by the question “Have you had sick leave the last 12 months?,” if yes, they were asked “What is your total sick leave in the last 12 months?” with alternatives of more or less than 2 wks.

### Exposure measurements

To assess workers' physical behaviors and exposure to awkward postures during workdays, each participant was equipped with 3 AX3 accelerometers (Axivity Ltd., Newcastle upon Tyne, UK). These devices were attached to their dominant side, on the thigh, arm, and upper back, as work tasks are most likely performed using the dominant side. The accelerometers were worn for up to 7 consecutive days and were attached with double-sided adhesive tape (3M; Witre, Halden, Norway) and waterproof medical tape (Opsite Flexifix). The accelerometers were initialized to measure at 25 Hz with a range of ±8 g using OmGui (version 1.0.0.43; Axivity Ltd.). The combination of the 3 accelerometers allows sensitive and specific measurements of lying, sitting, standing, walking, running, stair-climbing, cycling, or rowing, as well as different degrees of arm elevation and trunk forward bending (Korshøj et al. 2014; Skotte et al. 2014; Stewart et al. 2018). Participants were given a paper activity diary to register the time of getting up in the morning, arriving at work, finishing work, and going to sleep.

### Pain assessment

Participants assessed the intensity of NSP and LBP at the end of each workday by the following questions on the activity diary: “How much pain did you have in the shoulder/neck at the end of this working day?” and “How much pain did you have in the lower back at the end of this working day?”. The participants recorded the pain on a Numeric Pain Rating Scale (NPRS) with 11 integers from 0, representing no pain to 10 representing severe pain (Jensen et al. 1999). The NPRS is valid and reliable (Jensen et al. 1999) and is widely used for assessing musculoskeletal pain (Hoogendoorn et al. 2002; Williamson and Hoggart 2005; Hallman et al. 2019; Gupta et al. 2020).

### Data processing

**Fig. 1.**
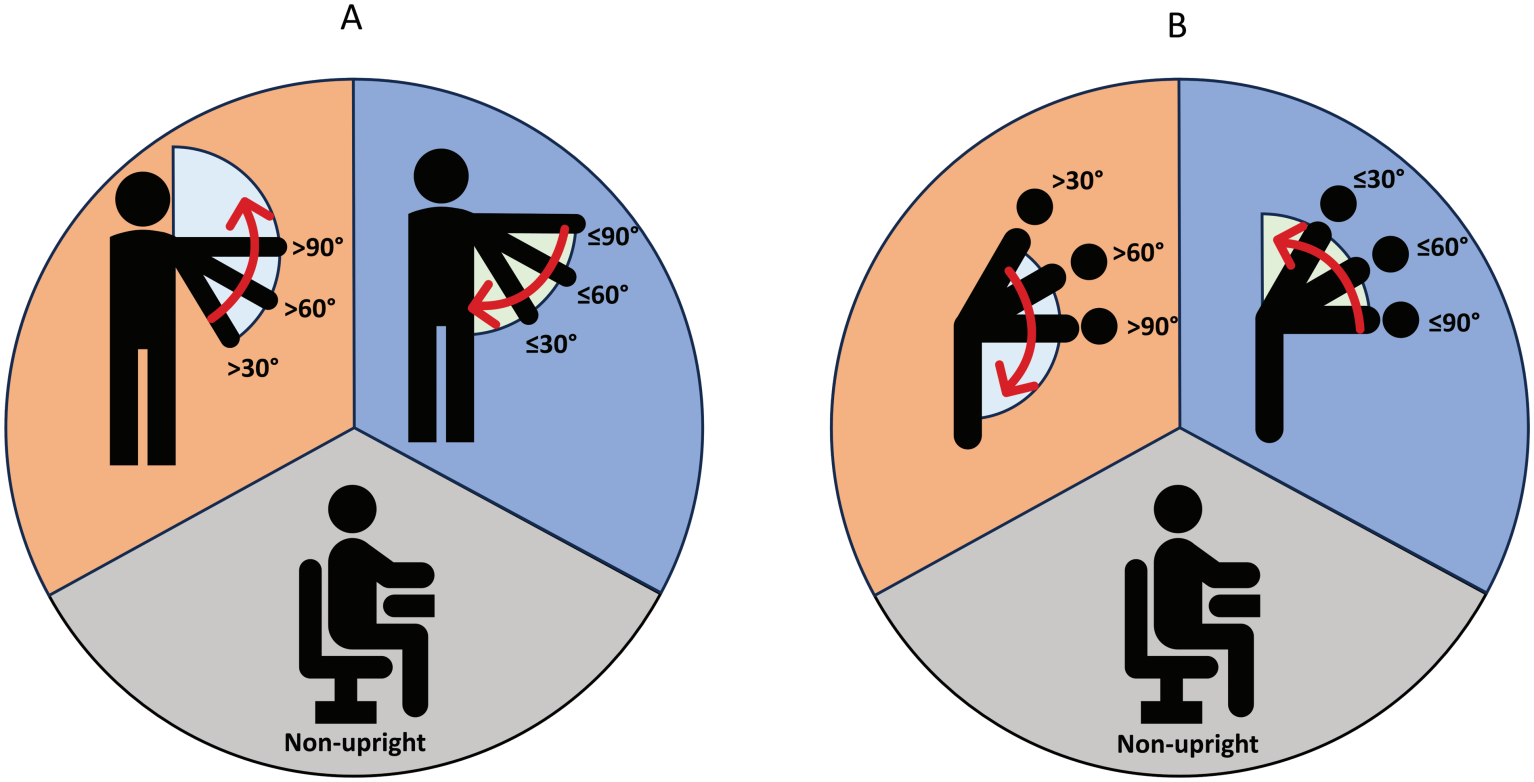
Illustration of how compositions of time below cutoff and above cutoff were constructed for arm elevation (A) and trunk inclination (B). Nonupright (gray) includes all time spent nonupright, regardless of arm elevation and trunk inclination. Below cutoff (blue) includes cutoff and all degrees below while upright, while above cutoff (orange) includes greater than cutoff degrees while upright.

Accelerometer data were processed into time spent in postures and behaviors using Acti4, a custom MATLAB software (developed by The National Research Centre for the Working Environment, Copenhagen, Denmark, and The Federal Institute for Occupational Safety and Health, Berlin, Germany (Korshøj et al. 2014; Skotte et al. 2014; Stemland et al. 2015)). Time spent in upright posture (standing, walking, running, stair walking) was then separated into time in awkward postures (i.e. above cutoffs for arm elevation and trunk inclination), and nonawkward posture (i.e. cutoff or below for arm elevation and trunk inclination). Nonupright time (i.e. sitting and lying down) was not divided into time with arm elevation and trunk inclination (Fig. 1). The information from the activity diaries was used to separate activity into periods of work, leisure, and sleep. To ensure accelerometer data used in the analysis represented complete workdays, workdays with <4 h of wear time were removed. Furthermore, we removed workdays that were clear outliers and/or if accelerometers were not worn correctly.

### Log-ratio coordinate representation of posture time compositions

Data describing the partition of time across a range of behaviors over an observation period correspond to so-called compositional data (Chastin et al. 2015; Dumuid et al. 2020). The fractions of time allocated to each behavior are intrinsically co-dependent and convey relative information. Hence, studying and interpreting them in isolation hinders the understanding of their potential interactions and combined effects on a response variable of interest (Chastin et al. 2015; Gupta et al. 2020). We defined 6 posture time compositions, based on cutoffs used in previous studies investigating arm elevation and trunk forward bending (arm elevation cutoffs of 30°, 60°, and 90°, and trunk inclination cutoffs of 30°, 60°, and 90°) (Gupta et al. 2021, 2022). Two observations, on 2 different days, contained a zero value for the above 90° trunk inclination behavior and multiplicative simple replacement was used to impute them (Palarea-Albaladejo and Martín-Fernández 2015; Rasmussen et al. 2020). By following the compositional data analysis (CoDA) approach (Pawlowsky-Glahn et al. 2015), the 6 three-posture compositions were expressed in the form of 2 isometric log-ratio (ilr) coordinates for statistical analysis, so that these represented normalized trade-offs or balances between the time spent in different posture behaviors. Using the case of 60° arm elevation for illustration, the following equations show the ilr coordinates computed in this study to represent the information from each individual:


$$\begin{array}{l} ilr_{1}=\sqrt{\frac{2}{3}}\ln\left(\frac{arm\;elevation>60^{\circ }upright}{\sqrt[{2}]{arm\;elevation\leq 60^{\circ }\;upright\times nonupright}}\right) \end{array}$$

and


$$\begin{array}{l} ilr_{2}=\sqrt{\frac{1}{2}}\ln\left(\frac{arm\;elevation\leq 60^{\circ }upright}{nonupright}\right) \end{array}$$

Thus, the coordinate ilr_1_ represents the time spent upright with arm elevation above 60° relative to the geometric mean of time spent upright with arms at 60° or below and time spent nonupright (i.e. sitting), and ilr_2_ represents the time upright spent with arms at 60° or below relative to the time spent nonupright. Analogous ilr coordinates were then computed for the remaining arm elevations and trunk inclinations considered.

### Statistical analysis

Exposure to awkward postures was summarized using the compositional geometric mean according to its relative nature (i.e. computing the vector consisting of the geometric means of each behavior), adjusted to add up to 100% and taking the average workday length as reference. Moreover, to describe the distribution of NSP and LBP, scores were summarized using ordinary arithmetic means and standard deviations, and incidence by pain grade: no pain (pain score 0), mild pain (1–3), moderate pain (4–6), and severe pain (7–10) (Brown et al. 2012).

To investigate the association between exposure to awkward postures during the workday, and NSP and LBP score (from 0 to 10) at the end of the workday, we defined 6 models, one for each arm elevation cutoff (30°, 60°, and 90°) and trunk inclination cutoff (30°, 60°, and 90°). The focus was on total time in awkward postures, we therefore did not differentiate between occupational groups, who might perform different tasks. All occupational groups were therefore included equally in the analysis. Poisson generalized linear mixed models (GLMMs) were fitted to, respectively, the NSP and LBP scores, including the ilr coordinates representing the awkward posture composition as exposure variables, individual ID as a random effect to account for repeated pain measures amongst participants, and the logarithm of total time at work as an offset, to control for different duration of working days. While we had data on a day-to-day basis, these were aggregated and used jointly in the modeling, considering random effects to account for the fact that they were repeated measures on the participants. Hence, the analysis refers to overall associations. Moreover, the models were adjusted for relevant covariates including age, gender, and BMI. These models adequately accounted for the Poisson count process determining the pain scores as response variables. Statistical assessment concluded no zero inflation nor overdispersion issues. The likelihood ratio test was used to assess the statistical significance of the association between the awkward posture composition and LBP and NSP scores. Finally, the fitted models were used in isotemporal substitution analysis to estimate expected values (marginal means) of the LBP and NSP scores (and associated 95% confidence intervals) in response to time reallocations between posture behaviors, using the mean posture composition as baseline. Namely, the analysis focused on one-to-one time reallocations of 1 to 20 min from either nonupright or below-cutoff postures to above-cutoff postures as the most relevant reallocations in the context of this study. The decision to limit the reallocation to 20 min was based on uncertainty around predictions increasing with time reallocated. Additionally, predicting far beyond realistic exposures—especially, in the case of 60° and 90°—was not considered prudent.

All statistical analyses were conducted on the R system for statistical computing v4.2.2 (R Foundation for Statistical Computing, Vienna, Austria). Statistical significance was concluded at the usual 5% significance level.

## Results

**Fig. 2.**
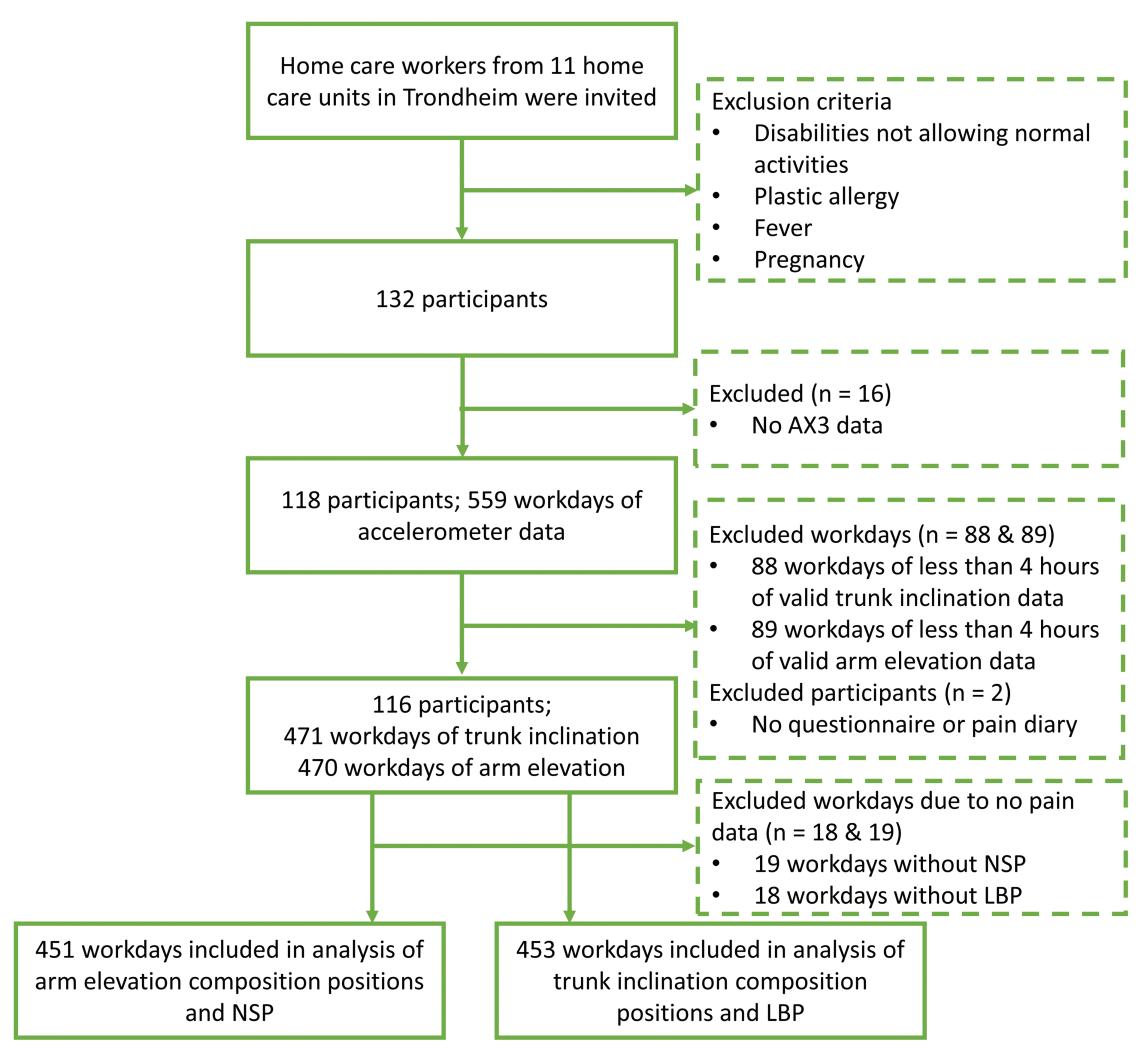
Flow chart of participants.

A total of 132 participants from 11 home care units in Trondheim, Norway, were enrolled in the study. After exclusion due to missing data, 116 participants were included for analysis of NSP and 115 for analysis of LBP (Fig. 2). Further, descriptive statistics, including demographics, health, and work status are displayed in Table 1. The average age of participants was 32.3 years, with 78.1% female and 21.9% male, and the average BMI was 27.1 kg/m^2^. Most of the participants had taken sick leave in the last 12 months (81.7%).

**Table 1.** Descriptive statistics of participants included in the study.

Demographic characteristics	%	*N*	Mean (SD)
Age (years)		116	32.3 (10.5)
Gender		116	
Female	78.1	89	
Male	21.9	25	
Body mass index (kg/m^2^)		116	27.1 (5.3)
Marital status		113	
Not married/living alone	65.4	74	
Married/partner	34.6	39	
Origin		115	
Scandinavian countries	92.2	106	
Non-Scandinavian countries	7.8	9	
Job title		115	
Nurse	34.8	40	
Occupational therapist	10.4	12	
Social worker	8.7	10	
Health assistant	35.6	41	
Other	10.4	12	
Sick leave in the last 12 months	81.7	94	
<2 weeks	57.4	54	
>2 weeks	42.6	40	

### Work exposures and musculoskeletal pain

A total of 116 participants had complete questionnaire data 451 working days of valid accelerometer data with NSP, whereas 115 participants had 453 days of valid trunk accelerometer data with LBP. The mean daily working time was 459 min and the mean number of valid working days was 3.9. Compositional geometric means of different exposures at work normalized to the average workday length are presented in Table 2. The mean NSP score was 1.64 (SD 2.2), and 54.9% of the working days reported NSP, of which 37.1% were mild pain, 13.1% were moderate pain, and 4.7% were severe pain. The mean LBP score was 1.4 (SD 1.9) and 55.5% of the working days reported LBP; of which, 41.4% were mild pain, 11.9% were moderate pain, and 2.2% were severe pain.

**Table 2.** Compositional geometric mean and percentage work exposures of study participants.

Posture composition	Behaviour	Mean (min)	Percentage
	Total time	459.0	100
Arm elevation 30°	Arm elevation >30°	49.8	10.8
	Arm elevation ≤30°	151.9	33.1
	Nonupright	257.3	56.1
Arm elevation 60°	Arm elevation >60°	6.6	1.4
	Arm elevation ≤60°	195.9	42.7
	Nonupright	256.5	55.9
Arm elevation 90°	Arm elevation >90°	1.2	0.3
	Arm elevation ≤90°	201.6	43.9
	Nonupright	256.2	55.8
Trunk forward bending 30°	Trunk forward bending >30°	33.0	7.2
	Trunk forward bending ≤30°	168.1	36.6
	Nonupright	258.0	56.2
Trunk forward bending 60°	Trunk forward bending >60°	13.5	2.9
	Trunk forward bending ≤60°	188.2	41.0
	Nonupright	257.2	56.1
Trunk forward bending 90°	Trunk forward bending >90°	2.7	0.6
	Trunk forward bending ≤90°	199.8	43.5
	Nonupright	256.5	55.9

Mean values of exposure to different postures based on 451 working days from arm accelerometer records and 453 working days from trunk accelerometer records. Trunk forward bending and arm elevation at >30°, >60°, >90° and ≤30°, ≤60°, ≤90° are in upright posture which includes standing still, moving, walking, running, and stair-climbing.

### Association between arm elevation and NSP

**Fig. 3.**
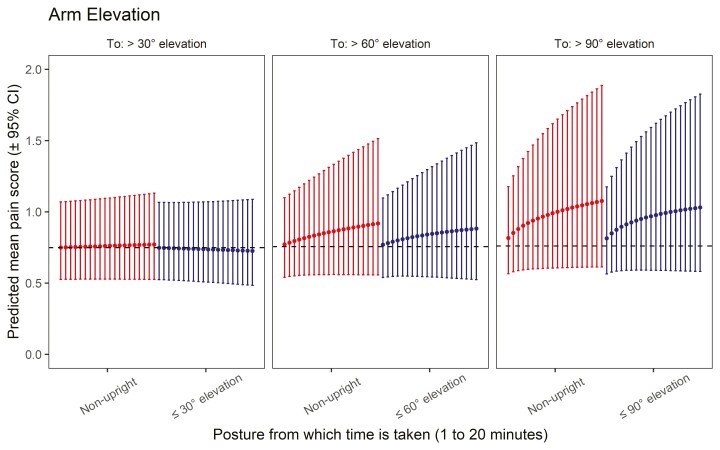
Results from isotemporal substitution analysis from Poisson GLMM fits showing the expected association between arm elevation and NSP. From left to right, the points represent predicted mean pain scores (± 95% confidence interval) from the reallocation of +1 min (1 to 20 min) from nonupright (red/left) and below-cutoff elevation (right/blue) to above-cutoff elevation (30°, 60°, and 90°). The gray line represents the predicted pain score for the mean composition.

The posture compositions regarding 30°, 60°, and 90° arm elevations were all significantly associated with NSP (*P* = 0.009, 0.006, and 0.003, respectively). The results from the isotemporal substitution analysis are displayed in Fig. 3. They illustrate the impact of reallocating up to 20 min to the 3 above-cutoff postures (i.e. >30°, >60°, and >90° arm elevation) from the remaining postures at the mean posture composition. The predicted pain for the mean posture compositions were 0.748, 0.756, and 0.760, respectively. Small and nonconsistent relative change in NSP is predicted when reallocating time from either nonupright or ≤30° arm elevation to >30° arm elevation. For instance, 0.7% and −0.7% mean changes in the NSP score are, respectively, predicted when reallocating 5 min (relative to pain score at the mean posture composition). However, when reallocating to >60° arm elevation, a more consistent positive trend is predicted. Thus, adding 5 min from nonupright or ≤60° arm elevation to >60° arm elevation is associated with predicted mean increases in the NSP score of 7.8% and 6.8%, respectively. Note that this positive association becomes steeper in the case of transfers to 90° arm elevation, e.g. implying that reallocations of 5 min to >90 arm elevation from nonupright or ≤90° arm elevation are linked to mean increases of 21.4% and 19.9% in NSP score, respectively. Note that there is a relatively large degree of uncertainty around these estimates as stressed by the width of the 95% confidence intervals. A table detailing all percentage changes, point predictions, and associated 95% confidence intervals can be found in Supplementary File 1.

### Association between trunk forward bending and LBP

**Fig. 4:**
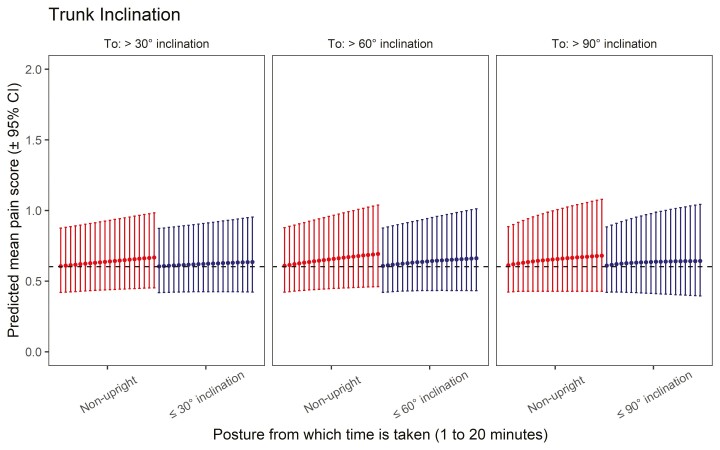
Results from isotemporal substitution analysis from Poisson GLMM fits showing the expected association between trunk inclination and lower back pain. From left to right, the points represent predicted mean pain scores (± 95% confidence interval) from the reallocation of +1 min (1 to 20 min) from nonupright (left/red) and below-cutoff inclination (right/blue) to above-cutoff inclination (30°, 60°, and 90°). The gray line represents the predicted pain score for mean composition.

The posture compositions regarding 30°, 60°, and 90° trunk forward bending were significantly associated with LBP (*P* ≤ 0.001, ≤0.001, and 0.001, respectively). The predicted pain for the 3 mean forward bending compositions was 0.602. Figure 4 summarizes the results from the isotemporal substitution analysis and illustrates how the reallocation of time from nonupright and below-cutoff to above-cutoff trunk forward bending is associated with increasing LBP by the fitted models. Thus, an increased mean LBP score is predicted when reallocating 5 min to >30° trunk forward bending from nonupright or ≤30° forward bending (respectively, 1.8% and 3.0% change relative to the mean posture composition). Analogously, increased mean LBP is predicted when reallocating 5 min to >60° trunk forward bending from nonupright or ≤60° forward bending (4.7% and 3.5%, respectively); or when reallocating 5 min to >90° trunk forward bending from nonupright or ≤90° forward bending (5.5% and 4.0%, respectively). Again, the 95% confidence intervals suggest a non-negligible degree of uncertainty around the estimates. A table detailing all percentage changes, point predictions, and associated 95% confidence intervals can be found in Supplementary File 1.

## Discussion

This study is the first to investigate the relationship between device-measured arm elevation and trunk forward bending and musculoskeletal pain in home care workers. We observed a positive association between NSP and time with arm elevation at >60° and >90°, with increased NSP score being associated with higher degrees of arm elevation. While reallocating time from nonupright to >30° arm elevation was associated with increased pain, reallocating time from upright with arms elevated ≤30° to >30° was associated with decreased pain. Reallocating time to trunk forward bending of 30°, 60°, and 90° was associated with an elevated LBP score, but the more extreme degrees of forward bending (60° and 90°) were not associated with a substantial additional LBP score, compared with 30°. For all outcomes, the isotemporal substitution analysis indicated that reallocating time from nonupright time is associated with a greater increase in pain score, compared with reallocating from below-cutoff upright time.

The difference in predicted pain scores based on reallocation of time from nonupright versus below-cutoff can likely be attributed to the inclusion of standing time in the below-cutoff time. Standing time is in itself associated with increased NSP and LBP (Coenen et al. 2018). The difference is most evident in arm elevation above 30° being negatively associated with NSP when time is reallocated from ≤30° postures. This might suggest that arm elevation of >30° does not add sufficient additional risk to outweigh the negative effects of standing. Overall, our findings support the hypotheses that arm elevation at work is associated with increased NSP and trunk forward bending with LBP.

Musculoskeletal pain is a multifaceted and complex issue. Our study addresses only a fraction of the potential causes of pain. Additional physical work exposures that may interact with awkward postures, but are not covered in the analysis, include forces exerted (i.e. the weight lifted by workers while in awkward postures) (Swain et al. 2020) velocities of movements (Arvidsson et al. 2021) and the temporal pattern (i.e. prolonged periods versus several shorter bouts) (Hanvold et al. 2012). Moreover, in accordance with the biopsychosocial model, psychological and social factors also play a crucial role in musculoskeletal pain (Niedhammer et al. 2021). Together, these elements and several others, not examined in our study, constitute the total work exposures leading to musculoskeletal pain.

Few studies have used technical measurements to investigate the association between arm elevation in upright postures and pain, but our results are consistent with most of the previous studies. A study by Hanvold et al. (2015) reported a positive association between arm elevation and NSP for women but did not find any association for men. However, a study by Svendsen et al. (2004) found a positive association in male participants. Similar to the current study, Merkus et al. (2021) used CoDA to analyze arm elevation and NSP after a 2-year follow-up, but found no association. In contrast, a study by Koch et al. (2017) suggested a trend of negative association, which is not in line with the results of our study. Regarding trunk forward bending, our study is consistent with most reports, finding that trunk forward bending is associated with increased LBP score. A 2012 meta-analysis by Griffith et al. (2012) concluded that a forward-bent posture was related to LBP; however, none of the included studies used technical measures. Furthermore, an umbrella review by Swain et al. (2020) found conflicting evidence, where 4 of 5 included reviews showed reasonable evidence for an association between awkward postures and increased LBP. Of studies utilizing technical measures, a study by Lunde et al. (2019) using accelerometers found a positive association between trunk forward bending >30° in upright postures and an increase in LBP intensity in healthcare workers. However, not all studies confirm these findings, one study by Villumsen et al. (2015) using accelerometers indicated a tendency of negative association among blue-collar workers. Furthermore, some studies have found no association between accelerometer-assessed trunk forward bending and LBP (Jansen et al. 2004; Lagersted-Olsen et al. 2016).

The conflicting evidence regarding awkward postures and NSP and LBP might be caused by pain-avoidance behavior where workers avoid behavior involving awkward postures, such as forward bending and elevated arms, due to fear of pain (Thomas and France 2008). It can also be caused by healthy worker effect, as workers susceptible to musculoskeletal pain quits, while workers without musculoskeletal pain may endure employment for longer (Punnett and Wegman 2004). Mixed results could also stem from different focus, as many did not investigate arm elevation or forward bending in upright postures exclusively. Awkward postures while sitting are more likely supported (e.g. driving, office work) resulting in a limited strain on the neck/shoulder and lower back area, therefore resulting in different pain outcomes (Palm et al. 2018). Furthermore, we do not have information about what tasks are being performed while forward bent and arms elevated. Performing a low-effort procedure, in contrast to manually handling a patient, while in an awkward posture, likely has a different impact on musculoskeletal pain. The study population may also play an important role in producing conflicting results, as arm elevation and forward bending performed in home care work may not be equivalent to similar behavior within other context. Lastly, our study stands out in comparison to previous studies that did not measure pain at the end of several workdays and did not consider the compositional nature of the data. In addition, very few studies have used accelerometers, which eliminate recall bias and allow separating upright behavior, from nonupright.

### Implications

While we found positive associations between exposures to awkward postures and musculoskeletal pain, the effect sizes were modest. However, this study only measured one week, and when considering the cumulative exposure over a working life, modest changes may lead to larger consequences, as both forward bending and arm elevation are associated with increased risk of sickness absence (Gupta et al. 2021, 2022).

Our findings highlight the importance of safeguarding home care workers against increased risk of musculoskeletal pain associated with awkward postures. Nevertheless, it is essential to acknowledge that the nature of home care work, being conducted within residents’ homes, may present challenges in optimizing the work environment to facilitate better ergonomic postures. Consequently, a degree of arm elevation and forward bending inherently occurs during home care workday.

Tjøsvoll et al. (2022) reported a substantial variation in exposure to forward bending and arm elevation among home care workers in Norway. Considering our findings in light of these results suggests that some home care workers are at a notably higher risk of LBP and NSP. We, therefore, propose the development and implementation of interventions aimed at decreasing the inter-individual differences in exposure to awkward postures among home care workers. By addressing this variability and focusing on protecting the most highly exposed individuals, it is conceivable that such interventions could contribute to an overall reduction in the prevalence of musculoskeletal pain within this occupational group (Lohne et al. 2022).

### Strengths and limitations

First, the biggest strength of this study is the use of device-based measurements to record the arm elevation and trunk forward bending for several consecutive days, which provided accurate information on work exposures and excluded subjective biases. The second is the novel use of CoDA methods to explore the relationship between awkward postures at work and musculoskeletal pain. This treated time-use data synergically as parts of a whole, thus allowing us to explicitly consider the interplay between the time spent in different postures and the association of this with musculoskeletal pain. Lastly, using NPRS immediately after finishing work, and thus recording the current pain intensity, is another strength since it reduces the recall bias (Euasobhon et al. 2022).

Admittedly, this study also has limitations. First, this is a cross-sectional study investigating the association between awkward work postures and musculoskeletal pain, thus it cannot inherently differentiate the direction of any cause-and-effect relationship. However, reverse causation by which musculoskeletal pain might lead to awkward postures is regarded unlikely, since people suffering from musculoskeletal pain tend to avoid awkward postures (Thomas and France 2008; Koch et al. 2017). Including pain score at the start of the workday would allow for controlling reverse causation, and thereby strengthen the quality of the analysis. Second, a relatively small fraction of home care workers in Trondheim, namely 26.3%, participated in this study. Therefore, some selection bias may have occurred. Increasing the sample size in future studies would help addressing this issue. Thirdly, the quality of the study would be strengthened by the inclusion of work exposures such as force production, temporal pattern, and velocity of movements. Lastly, the participants had very little exposure to the more extreme awkward posture, e.g. 90° trunk forward bending and arm elevation. This limits the scope of the isotemporal substitution analysis conducted.

## Conclusions

This study revealed a positive correlation between arm elevation (>60° and >90°) in upright postures and NSP intensity in home care workers, with NSP intensity increasing more at higher arm elevations. Additionally, while trunk forward bending in upright postures was significantly associated with LBP, there was minimal increase beyond 30°. These findings should guide work pattern adjustments to mitigate musculoskeletal pain risks in home care workers.

## Supplementary material

Supplementary material is available at *Annals of Work Exposures and Health* online.

wxae027_suppl_Supplementary_File_1

## Data Availability

Data can be shared upon reasonable request to the corresponding author. Aggregated data for isotemporal substitution can be found in Supplementary material.

## Declaration

During the preparation of this work, the corresponding author (F.K.L.) used ChatGPT (openai.com) to improve readability and language. After using this tool, all authors reviewed and edited the content as needed and take full responsibility for the content of the publication.
